# Immunohistochemistry combined with NGS to assist the differential diagnosis of multiple primary lung cancer with lymph node metastasis: a case report

**DOI:** 10.3389/fonc.2023.1260759

**Published:** 2023-10-18

**Authors:** Chang Liu, Shuai Zhang, Hong Yang, Yu Bai, Yanru Shen, Yi Ren

**Affiliations:** ^1^ Department of Thoracic Surgery, Shenyang Tenth People’s Hospital, Shenyang Chest Hospital, Shenyang, China; ^2^ Department of Pediatric Urology, Dalian Women and Children’s Medical Center (Group), Dalian, Liaoning, China; ^3^ Department of Pathology, Shenyang Tenth People’s Hospital, Shenyang Chest Hospital, Shenyang, China; ^4^ Department of Thoracic Surgery, The Second Affiliated Hospital of Dalian Medical University, Dalian, China; ^5^ Medical Project, Berry Oncology Corporation, Fuzhou, Fujian, China

**Keywords:** multiple primary lung cancers, next-generation sequencing, immunohistochemistry, osimertinib, lymph node metastasis

## Abstract

In recent years, the incidence of synchronous multiple primary lung cancers (MPLCs) has gradually increased. Surgery is the preferred treatment for these patients. There are great differences in the driving genes between individual tumors in patients with MPLC, and tumors with targeted mutations do not represent other tumors, which challenges the selection of targeted therapies for patients with MPLC. Driving mutations in each lesion after surgery are crucial for establishing accurate pathological staging and subsequent treatment strategies. There are some mutated genes in the lymph nodes of postoperative metastatic MPLCs, and the tumor cell count/DNA concentration is low, which limits the next-generation sequencing (NGS) detection effect. A combination with immunohistochemistry to determine the source of metastasis may be a better choice. This study reports a rare case of lung cancer with double primary adenocarcinomas of the lung combined with 10 groups of lymph node metastases. The source of metastasis was identified using immunohistochemistry combined with NGS to guide postoperative adjuvant treatment. We hope that this case report can provide new ideas for the identification of MPLCs and assist in their diagnosis and individualized treatment. In addition, the combination specific immunohistochemistry and NGS seems to be an effective identification method. This approach can provide clinical benefits; however, this still requires further exploration through studies with large sample sizes.

## 1 Introduction

Multiple primary lung cancers (MPLCs) are defined as two or more primary lung cancers that occur in the same patient. Depending on the time of occurrence, they can be divided into synchronous and metachronous MPLCs (1). With the wide application of computed tomography and lung cancer screening, the incidence of MPLC in lung cancer patients is 0.2~8% (2). ACCP recommends surgery as the preferred treatment for MPLC; however, the optimal surgical strategy for MPLC remains controversial (3). The driver mutation of each lesion after surgery is important for establishing accurate pathological staging and subsequent treatment strategies. Targeted therapy has been successfully and widely used to treat lung cancers with driver gene mutations (4). The incidence of epidermal growth factor receptor (EGFR) mutations in patients with MPLC is high (5). This indicates that EGFR-tyrosine kinase inhibitors can play an active role in postoperative treatment. However, there are large differences in driver genes between individual tumors in patients with MPLC, and tumors with targeted mutations cannot represent other tumors, which complicates the choice of targeted therapy for patients with MPLC (6). In the case of multifocal lung cancers, discriminating MPLCs from intrapulmonary metastases remains a common dilemma in the clinical setting. The main reason for this difficulty in identification is that the histological types are identical in most patients with multifocal lung cancers, with adenocarcinoma being the most frequent (7). MPLC diagnosis and treatment have made great progress with the development of various technologies, especially next-generation sequencing (NGS) (7). However, detection requires a sufficient proportion of tumor cells or a high DNA concentration, limiting it to small biopsy samples (8). Immunohistochemistry (IHC) is a simple, rapid, mature, and routine method that is not affected by tumor volume and DNA degradation. Previous studies have confirmed that the mutation-specific antibody SP125 has a high specificity for lung adenocarcinoma *EGFR* L858R (9). At present, the identification of MPLC lymph node metastasis is still controversial. This existing knowledge gap can be addressed through the practices employed in the current case study. This study reports a rare case of lung cancer with double primary lung adenocarcinoma lesions and ten groups of lymph node metastases. The source of metastasis was identified via IHC combined with NGS detection to guide postoperative adjuvant therapy.

## 2 Case presentation

**Figure 1:**
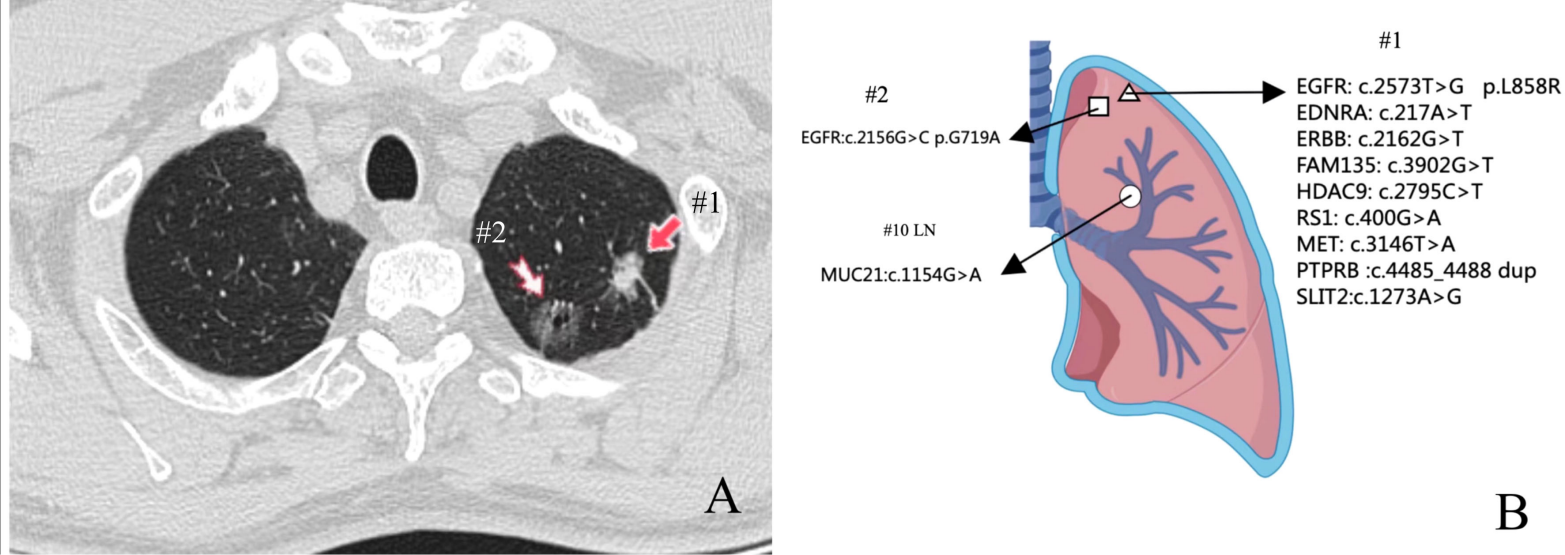
Location and next-generation sequencing (NGS) results of the three pulmonary lesions. **(A)** Chest computed tomography showed two ground-glass density nodules in S1 of the upper lobe of the right lung. **(B)** Genomic alterations detected via NGS analysis in three lesions.

**Figure 2:**
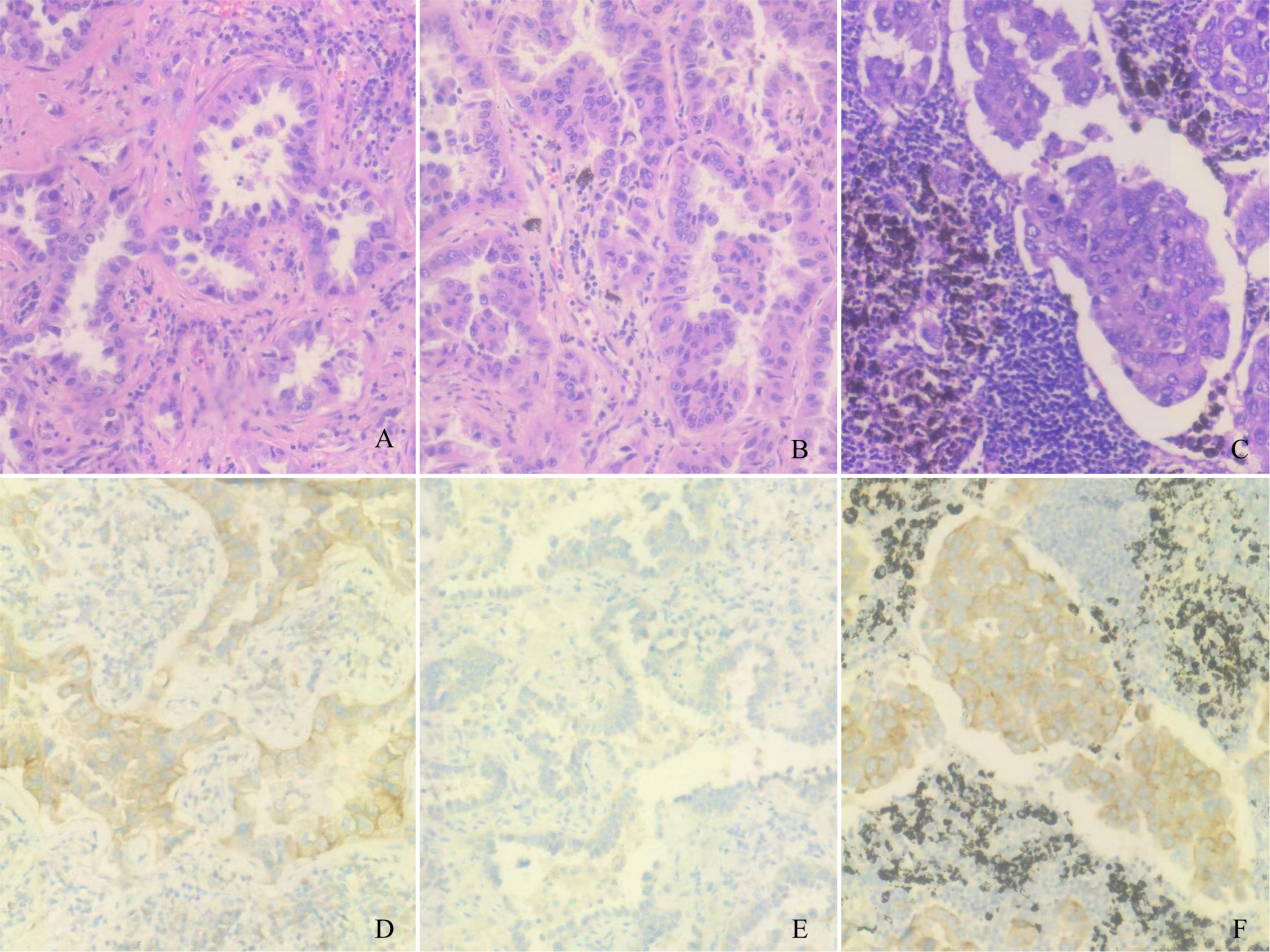
Pathological and immunohistochemical examination results. **(A)** Lymph node #1 hematoxylin-eosin staining, magnification: ×200. **(B)** Lymph node #2 hematoxylin-eosin staining, magnification: ×200. **(C)** Lymph node #10 hematoxylin-eosin staining, magnification: ×200. **(D)** Lymph node #1 SP125 positivity, magnification: ×200. **(E)** Lymph node #1 SP125 negativity, magnification: ×200. **(F)** Lymph node #10 SP125 positivity, magnification: ×200.

A 61-year-old man underwent chest computed tomography during a routine health examination. Chest computed tomography revealed two ground-glass density nodules in S1 of the upper lobe of the right lung. The larger nodule (#1) was an irregular, partially solid nodule of 17 mm, and the other nodule (#2) was a pure ground-glass nodule of 16 mm (**Figure 1A**). There was no hilar or mediastinal lymph node enlargement, and the patient had no obvious medical, occupational, or smoking history. Bone imaging and head magnetic resonance imaging were used to detect systemic metastasis; no extrapulmonary metastasis was observed, and no abnormalities were found in the blood cell count, tumor markers, or biochemical tests. The cardiopulmonary function was normal, and the patient underwent video-assisted thoracoscopic surgery for left upper lobectomy + lymph node dissection. The pathology of left upper lobe #1 was invasive adenocarcinoma of the acinar (80%), lepidic growth pattern (10%), and micropapillary architecture (10%). The pathology for #2 was invasive adenocarcinoma of the acinar (90%) and lepidic growth pattern type (10%), the 10^th^ group of lymph nodes with metastasis. To understand the mutations in #1 and #2 tumor samples and verify the metastatic source of lymph node #10, we obtained permission from the patient and used nanograms for gene analysis. This method aimed to identify 831 somatic variants of genes related to tumor occurrence and development and 148 genetic susceptibility-related germline variants. Somatic variants included *EGFR, KRAS, NRAS, BRAF, RET, PIK3CA*, and *TP53*, while germline variants included *APC, BRCA1, BRCA2, EpCAM*, and *MSH6*. Genetic analysis of the tumor samples was conducted by Beijing Genetron Health Technology Co., Ltd. using the Illumina Novaseq 6000 platform. Among the 831 genes tested, nodule #1 harbored mutations in *EGFR, EDNRA, ERBB3, FAM135 B, HDAC9, IRS1, MET, PTPRB*, and *SLIT2*. *EGFR* showed a missense mutation (C.2573T>G, p.L858R) in exon 21. For nodule #2, there was a missense mutation in *EGFR* (c.2156G>C p.G719A) in exon 18. *MUC21* mutation (c.1154G>A p.S385N) was present in lymph node #10 (**Figure 1B**). In view of the genetic testing results, we considered #1 and #2 to be double primary lesions. To verify the source of metastasis of lymph node #10, we performed SP125 IHC detection on lymph nodes #1, #2, and #10. We found that lymph nodes #1 and #10 were both positive for expression and considered lymph node #10 as a #1 lesion metastasis (**Figure 2**). The patient was considered to have *EGFR* (C.2573T>G, p.L858R) of exon 21 in both lymph nodes #1 and #10. Since February 2022, the patient has been taking osimertinib 80 mg/day, and has recovered well with no progression. This study was approved by the Clinical Trial Ethics Committee of Shenyang Tenth People’s Hospital, and informed consent was obtained from the patient.

## 3 Discussion

MPLCs are a common type of lung cancer, highlighting the complex biological characteristics of this malignancy, particularly lung adenocarcinoma. MPLC diagnosis and treatment remain controversial, and there is currently no diagnostic method to distinguish between multiple primary and metastatic diseases (10). With the development of NGS technology, using tumor driver gene mutations as biomarkers can help identify MPLC and intrapulmonary metastases in patients with lung adenocarcinoma (11). Previous studies have shown that the frequency of gene mutations mainly determines the likelihood of identical gene mutations occurring between tumor pairs. If multiple tumors have the same mutated gene, a lower mutation frequency of that gene indicates that the tumors belong to the same clone origin, revealing a greater diagnostic value. In contrast, patients with the same high frequency of gene mutations require caution in diagnosing whether their clones originate from the same source (12).

This report shows a very high driving mutation rate of MPLC, especially the *EGFR* mutation, indicating an opportunity for targeted therapy in MPLC management. Although there are good examples of targeted therapies for patients with non-small cell lung cancer, the clinical situation of patients with MPLC is unique, because a lesion carrying a targeted mutation does not represent all lesions. A difference rate of driving gene mutations in MPLCs as high as 92% was previously reported (13, 14), which led to different responses of patients with MPLC to targeted therapy, presenting a major challenge to the application of targeted drugs in MPLC treatment. There are very few mutated genes in metastatic lymph nodes, which may only be related to metastasis of the primary lesion. The mutated genes in the metastatic lymph nodes are similar to those in the corresponding primary tumor, indicating the clonal evolution of the tumor (15). Here, we report a rare case of two primary adenocarcinomas of lung lesions, with 10 groups of lymph node metastases. In the left upper lobe lesion #1, *EGFR* showed a missense mutation (C.2573T>G, p.L858R) in exon 21. In #2, *EGFR* showed a missense mutation (c.2156G>C p.G719A) in exon 18; a *MUC21* mutation (c.1154G>A p.S385N) was present in lymph node #10. SP125 IHC tests were positive in #1 and #10, while #2 was negative. It was considered that lymph node #10 is homologous to lesion #1. The patient was diagnosed with stage T1bN1M0 IIA for #1 and stage T1bN0M0 IB for #2. The patient was treated with 80 mg/day oral osimertinib as an adjuvant targeted therapy, which was well tolerated.SP125 exhibits a high specificity of 89.7% for the L858R mutation, but its sensitivity is relatively low at 80.4% (10). Because of the relatively low sensitivity of SP125, not all patients with mutated EGFR were detected.

This study provides novel insights for the diagnosis and treatment of patients with synchronous MPLC with lymph node metastasis. Owing to the molecular biology differences in synchronous MPLC, NGS technology should be used to detect the molecular biology of patient lesions to guide treatment. When the diagnosis of metastatic lymph node via NGS is unclear, the origin of the primary lesion can be determined via IHC. Further research is needed on application scenarios based on IHC and NGS detection. A large-scale prospective study should be conducted to establish a more powerful diagnostic examination that combines radiology, histopathology, and molecular examination to verify our conclusions.

## Data availability statement

The original contributions presented in the study are included in the article/supplementary material. Further inquiries can be directed to the corresponding author.

## Ethics statement

The studies involving humans were approved by Ethics Committee of Shenyang Tenth People’s Hospital. The studies were conducted in accordance with the local legislation and institutional requirements. The participants provided their written informed consent to participate in this study. Written informed consent was obtained from the individual(s) for the publication of any potentially identifiable images or data included in this article.

## Author contributions

CL: Writing – original draft. SZ: Data curation, Writing – review & editing. HY: Investigation, Writing – original draft. YB: Conceptualization, Writing – original draft. YS: Software, Writing – original draft. YR: Conceptualization, Writing – original draft.
